# A Holistic Approach to Urban and Children’s Mental Health

**DOI:** 10.1093/eurpub/ckac129.295

**Published:** 2022-10-25

**Authors:** P Van Den Hazel, G Ristovska, A Sandionigi, M Weber, S Benz

**Affiliations:** 1 INCHES, Ellecom, Netherlands; 2 Institute of Public Health of Republic of North Macedonia, Skopje, Republic of North Macedonia; 3 City of Utrecht, Utrecht, Netherlands; 4 Quantia Consulting srl, Milan, Italy; 5 Centre for Applied Psychology, Environmental and Social Research ZEUS, Hagen, Germany

## Abstract

**Background:**

EU data show that there is a continuous increase in mental health problems in children. The multiple exposures in the physical and social domain influence the internal exposome and the final health outcomes. The Equal-Life project research focuses on the exposome (physical, social and internal) and mental health of children and adolescents.

**Methods:**

Literature reviews on children's mental health and a total of physical and social exposures from 9 different European cohorts are used to set a framework of data interpretation. In addition, information gathered from 3 surveys/65 interviews with stakeholders from different fields was used to look for a transdisciplinary framework for analysis of the cohort data. The approach develops (in a co-design approach) tools that help stakeholders to collect and organize the information necessary to make decisions. The exposome is as if it were a multilayer system and requires approaches that are able to manage the interconnections between the various variables involved in the field of children's mental health.

**Results:**

The main points extracted are an early life exposure to urban planning related factors (noise/air pollution, traffic), social factors (family relations, stress), biomarkers related to environmental and social exposures. Concerning the life course approach, vulnerable settings were identified such as schools, neighbourhoods, accessibility to green restorative spaces. These ingredients are the basis of interaction between scientists and community stakeholders and from the policy domains to find and interpret the scientific results for the implementation of protective and promotive policies in cities.

**Conclusions:**

Transdisciplinary approach is necessary for management and developing strategies for solving mental health problems in urban spaces when considering all kind of exposures. The development of tools through co-design sessions with different stakeholders gives important contributions for this goal.

**Key messages:**

• Development of interventions and policies for better mental health of children and adolescents should be based on available evidence for meaning of external and social exposome.

• The main outcome of Equal-Life is to develop tools where stakeholders have access and opportunity to use it for solving mental health issues at national or local level.

